# Genetic Variants in Protein Tyrosine Phosphatase Non-Receptor Type 23 Are Responsible for Mesiodens Formation

**DOI:** 10.3390/biology12030393

**Published:** 2023-03-01

**Authors:** Ploy Adisornkanj, Rajit Chanprasit, Steven Eliason, Juan M. Fons, Worrachet Intachai, Sissades Tongsima, Bjorn Olsen, Stefan T. Arold, Chumpol Ngamphiw, Brad A. Amendt, Abigail S. Tucker, Piranit Kantaputra

**Affiliations:** 1 Center of Excellence in Medical Genetics Research, Faculty of Dentistry, Chiang Mai University, Chiang Mai 50200, Thailand; 2 Division of Pediatric Dentistry, Department of Orthodontics and Pediatric Dentistry, Faculty of Dentistry, Chiang Mai University, Chiang Mai 50200, Thailand; 3 Dental Department, Wiang Kaen Hospital, Wiang Kaen, Chiang Rai 57310, Thailand; 4 Department of Anatomy and Cell Biology and the Craniofacial Anomalies Research Center, The University of Iowa, Iowa City, IA 52242, USA; 5 Centre for Craniofacial and Regenerative Biology, King’s College London, Floor 27 Guy’ Hospital, London Bridge, London SE1 9RT, UK; 6 National Biobank of Thailand, National Science and Technology Development Agency, Thailand Science Park, Pathum Thani 12120, Thailand; 7 Department of Developmental Biology, Harvard School of Dental Medicine, Harvard University, Boston, MA 02115, USA; 8 Computational Bioscience Research Center, Biological and Environmental Science and Engineering, King Abdullah University of Science and Technology, Thuwal 23955-6900, Saudi Arabia; 9 Center for Structural Biology, National Institute of Health and Medical Research, National Centre for Scientific Research, University of Montpellier, 34090 Montpellier, France; 10 Iowa Institute of Oral Health Research, University of Iowa, Iowa City, IA 52242, USA

**Keywords:** extra tooth, *PTPN23*, mesiodentes, mutation, phosphatase, protein expression, RNA expression, supernumerary tooth

## Abstract

**Simple Summary:**

A mesiodens is an extra tooth located in the midline of the upper jaw. To investigate the genetic cause of mesiodens, clinical and radiographic examination were performed on 23 family members of a two-generation Hmong family. Whole exome or Sanger sequencing were performed in 22 family members. We found an extremely rare mutation (c.1807G>A;p.Glu603Lys) in the *PTPN23* gene in seven family members who had mesiodens. However, six family members who did not have mesiodens also carried the mutation. The mode of inheritance appears to be autosomal dominance with incomplete penetrance (53.84%). The finding of a *PTPN23* mutation as a cause of mesiodens phenotype is supported by the findings of two additional rare *PTPN23* mutations in two unrelated Thai patients with mesiodens. *PTPN23* is a regulator of endosomal trafficking functioning to move activated membrane receptors, such as EGFR, from the endosomal sorting complex for multivesicular body biogenesis, lysosomal degradation and subsequent downregulation of receptor signaling. Our immunohistochemical study and RNAscope on developing mouse embryos showed broad expression of *PTPN23* in oral tissues, while immunofluorescence showed that EGFR was specifically concentrated in the midline epithelium. Our study showed for the first time that genetic variants in *PTPN23* caused reduced phosphatase activity, disrupted midline signaling, and subsequent mesiodens formation.

**Abstract:**

A mesiodens is a supernumerary tooth located in the midline of the premaxilla. To investigate the genetic cause of mesiodens, clinical and radiographic examination were performed on 23 family members of a two-generation Hmong family. Whole exome sequencing (WES) or Sanger sequencing were performed in 22 family members and two unrelated Thai patients with mesiodens. WES in the Hmong family revealed a missense mutation (c.1807G>A;p.Glu603Lys) in *PTPN23* in seven affected members and six unaffected members. The mode of inheritance was autosomal dominance with incomplete penetrance (53.84%). Two additional mutations in *PTPN23*, c.2248C>G;p.Pro750Ala and c.3298C>T;p.Arg1100Cys were identified in two unrelated patients with mesiodens. *PTPN23* is a regulator of endosomal trafficking functioning to move activated membrane receptors, such as EGFR, from the endosomal sorting complex towards the ESCRT-III complex for multivesicular body biogenesis, lysosomal degradation, and subsequent downregulation of receptor signaling. Immunohistochemical study and RNAscope on developing mouse embryos showed broad expression of *PTPN23* in oral tissues, while immunofluorescence showed that EGFR was specifically concentrated in the midline epithelium. Importantly, *PTPN23* mutant protein was shown to have reduced phosphatase activity. In conclusion, mesiodens were associated with genetic variants in *PTPN23*, suggesting that mesiodens may form due to defects in endosomal trafficking, leading to disrupted midline signaling.

## 1. Introduction

A mesiodens, a term first used by Bolk in 1917, is a supernumerary tooth located in the midline of the premaxilla region [1]. The prevalence of mesiodens ranges from 0.1 to 5%, depending on the population studied [2,3,4,5,6,7,8,9]. Both the highest and lowest prevalences were reported in the Turkish population, with 5.04% [8] and 0.1% [9], respectively (Table 1). Males are affected approximately twice as often as females [3,5,6,7,8,10,11,12], especially in Japan (2.8:1) [13] and Switzerland (2.6:1) [14].

Most mesiodens are found as single supernumerary teeth with a vertical orientation; however, mesiodens may appear as double or multiple (mesiodentes) in different orientations (Table 2) [3,5,6,7,8,9,11,12,13,15]. Interestingly, more than 53% and up to 78.8% of mesiodens do not erupt into the oral cavity; therefore, radiographic examination is required in order to make a diagnosis and identify the orientation of mesiodens for treatment planning [2,3,4,5,6,7,8,9,10,13,14]. Mesiodens are classified according to their morphology: conical, tuberculate, and supplemental type [16]. Mesiodens do not exfoliate and are, therefore, considered permanent teeth.

Several hypotheses have been proposed as to the etiology of mesiodens, including atavism, dichotomy, and hyperactivity of the dental lamina, with genetic factors believed to be important [17,18]. The occurrence of mesiodens in siblings [11,19,20,21,22,23,24], in family members of more than one generation [24,25,26,27], and in monozygotic twins support the role of genetic factors in the pathogenesis of mesiodens. Previous studies in monozygotic twins found five pairs of concordant, four pairs of mirror imaged discordant, and eleven pairs of discordant mesiodens [28,29,30,31,32,33,34,35,36,37,38,39,40]. Mesiodens are observed to skip a generation in affected families, favoring an autosomal dominant mode of inheritance with incomplete penetrance (Table 3) [27].

Notably, mesiodens have been reported in a number of patients with Nance–Horan syndrome [41,42,43,44] and holoprosencephaly [45]. Recently genetic variants in the WNT/β-catenin signaling pathway, including *LRP4*, *LRP5*, *LRP6*, *WLS*, and *DKK1*, have been demonstrated to be implicated in mesiodens phenotype [46,47,48,49,50]. The objective of our study was to investigate the genetic etiology of mesiodens in a two-generation Hmong family.

We performed whole exome sequencing (WES) in a large Hmong family affected with mesiodens and two additional unrelated Thai patients. Three rare variants in protein tyrosine phosphatase non-receptor type 23 (*PTPN23*; MIM 606584), a regulator of endosomal trafficking, were identified as causes of the mesiodens phenotype.

## 2. Materials and Methods

Ethical approval: This study was approved by the Human Experimentation Committee of the Faculty of Dentistry, Chiang Mai University (no. 71/2020) and was performed in accordance with the ethical standards of the Declaration of Helsinki. Written informed consent was obtained from all participants or from a legal guardian of children younger than 18 years old.

### 2.1. Clinical Examination, Sample Collection, and DNA Extraction

Clinical and radiographic examinations were performed on 23 family members of a Hmong family and two unrelated Thai patients with mesiodens.

Depending on the availability, either saliva or blood was used as a source of genomic DNA. Saliva was collected according to the Oragene DNA OG-575 kit (DNA Genotek Incorporated, Ottawa, ON, Canada). Genomic DNA was extracted and purified following the prepIT L2P reagent protocol (DNA Genotek Incorporated, Ottawa, ON, Canada). When blood was used, collection of 4 mL of blood in EDTA tubes was performed for some family members. The genomic DNA from whole blood was extracted according to the protocol of the QuickGene DNA whole blood kit (Kurabo industries Limited, Osaka, Japan).

The DNA samples were tested for protein contamination and concentration. Each DNA sample should contain double-strand DNA over 1 µg in quantity and 50 ng/µL in concentration. The DNA samples were then sent for WES (Macrogen Incorporated, Seoul, Republic of Korea).

### 2.2. Whole Exome Sequencing and Sanger Direct Sequencing

For 17 participants of the Hmong family (I-1, I-5, I-7, I-8, I-9, I-10, I-11, II-2, II-6, II-8, II-9, II-10, II-11, II-12, II-13, II-14, and II-15), the genomic DNA samples were subjected to exome capture using the SureSelect V6+UTR-post kit (Agilent Technologies, Santa Clara, CA, USA). The captured DNA underwent high throughput sequencing using the Illumina HiSeq platform (Illumina, San Diego, CA, USA). All prepared flow cells were run using paired-end 150-basepair reads. Reads were aligned to the human reference sequences (GRCh37) in the Burrows–Wheeler Alignment (BWA) tool version 0.7.17 to generated BAM files [51]. Using Genome Analysis Toolkit (GATK) version 3.8.1 software (Broad Institute, Cambridge, MA, USA) [52], both single nucleotide variants (SNVs) and small insertions/deletions (Indels) were identified in individual genome variant call format (gVCF) files. Then, the genotyped data of all samples were called by GenotypeGVCFs, and high-quality variants were obtained after a variant quality score recalibration (VQSR) step following the GATK best practice. All variants were assigned their pathogenic effect scores using the variant effect predictor (VEP) tool version 99 [53], as well as cross-checking against the database for nonsynonymous SNPs’ functional predictions (dbNSFP), version 3.5a [54]. General variant filtering was completed by (1) excluding the intron, intergenic and non-coding regions, (2) excluding synonymous variants, and (3) excluding common variants with allele frequencies of 1000 Genome project (http://www.1000genomes.org/ accessed on 8 January 2022), gnomAD exome and gnomAD genome (https://gnomad.broadinstitute.org/ accessed on 8 January 2022) greater than 0.01, 0.05, and 0.05, respectively.

Using combined VCFs, a combination file from 17 Hmong individual files with a list of all genotypes in separate columns was created. According to the pedigree, we hypothesized that the affected are inherited in an autosomal dominant mode, with some being non-penetrant; thereby, we specified filtering to identify candidate variants by conditioning seven affected members (I-5, II-8, II-9, II-11, II-12, II-14, and II-15) as heterozygous, eight unaffected members (I-1, I-8, I-9, I-11, II-2, II-6, II-10, and II-13) as wild-type or heterozygous (because not having mesiodens does not mean not having the genotype) and two unaffected and unrelated members (I-7 and I-10) as wild-type. After we identified a candidate gene, Sanger direct sequencing was performed on all DNA samples from the family using forward primer sequences: AGA CCC CAT TGG GAG ACT CG, and reverse primer sequences: AGG ACT GGG CAC TGA CTT TT). Sequencher 4.8 Sequence analysis software (Genecodes, Ann Arbor, Michigan, United States of America) was used to analyze variant presence.

Amino acid sequences of the protein in various vertebrate species were aligned with PLALINE multiple sequence alignment software (https://www.ibi.vu.nl/programs/pralinewww/ accessed on 8 January 2022) to calculate conservation consistency. MutationTaster (http://www.mutationtaster.org/ accessed on 8 January 2022), PolyPhen-2 (http://genetics.bwh.harvard.edu/pph2/ accessed on 8 January 2022), and sorting intolerant from tolerant (SIFT) software (http://provean.jcvi.org/index.php/ accessed on 8 January 2022) were used to predict the functional effects of the mutation.

### 2.3. Gene and Protein Expression

Expression of *PTPN23* was analyzed by RNAscope multiplex fluorescence assay (Advance cell diagnostics, ACD, a BioTechne brand) following the manufacturer’s instructions. RNAscope was used for *PTPN23* as it provides a very accurate and quantitative assessment of gene expression levels. *PTPN23* targets the availability of EGFR protein. Therefore, EGFR protein expression was analyzed using immunofluorescence to focus on the spatial distribution of this receptor. Sections were dewaxed 3 × 10 min in xylene and rehydrated in ethanol series (100%, 90%, 70%, 50%, and 30%) for a period of 2 min for each step. Antigen retrieval was performed in citric acid 0.01 M at 90 °C for 30 min or Tris EDTA at 90 °C for 30 min. Blocking was achieved in PBS with 0.025% Tween20, 1% BSA, and 10% serum. Primary antibody (anti-pEGFR) was applied with an overnight incubation at 4 °C. Secondary Goat anti-Rabbit biotin was used 1/800 (Dako, E0432) followed by Streptavidin–HRP (Abcam, ab64269). The color reaction was performed in TSA buffer (100 mM Borate buffer with 0.0003% hydrogen peroxidase) with Opal-570 1/300 (Akoya Bioscience, OP001003) for 10 min. Slides were mounted with fluoroshield with DAPI as a nuclei stain.

### 2.4. Computational Structural Analysis of Mutants

Sequence and sequence-related information were retrieved from the Uniprot database [55]. For structural analysis, crystallographic models for the Bro1 (PDB accession 5crv) and CC domain (PDB accession 5l mL) and the AlphaFold [56] theoretical model were used. Models were manually inspected, and mutations were evaluated using the Pymol program (http://pymol.org accessed on 8 January 2022). Linear sequence motifs were identified using the Eukaryotic Linear Motif (ELM) database [57].

### 2.5. PTPN23 Mutant Protein Stability

To evaluate the stability of *PTPN23* in cells, we purchased a *PTPN23* clone from Genescript in the plasmid pCDNA3.1(+). The c.1807 G>A; p.Glu603Lys; rs141113890 mutation was inserted by overlapping PCR and verified by sequence analysis. WT and Mutant *PTPN23* were transfected into dental and oral cell lines (HEK293, MDPC, and LS-8 cells) using Polyethylenimine (PEI). After transfection of HEK293 cells, lysates were made in RIPA buffer plus protease inhibitors and run on 8% SDS Page gels. Primary antibodies toward *PTPN23* (LS Bio, Inc., Seattle, WA, USA) and GAPDH (Santa Cruz Biotechnology, Dallas, TX, USA) were used for the detection of proteins. Identical lysates were run on different gels due to differences in acrylamide concentrations used to detect the proteins.

### 2.6. PTPN23 Mutant Phosphatase Activity

Phosphatase activity was measured using the EnzCheck Phosphatase assay kit (Molecular Probes) in samples without transfection, and those transfected with control DNA, WT *PTPN23* and Mutant *PTPN23*. Lysates were made in Reporter lysis buffer (Promega) and incubated with substrate DiFMUP and measured using an excitation of ~360 nm and an emission detection of 460 nm. Potato Acid Phosphatase, provided by the manufacturer, was used as a control. Each bar is an n = 3 on independent transfections, and each control was an independent dilution of the Potato Acid Phosphatase, n = 3. Results are expressed as a fold increase over untransfected, where values are set to 1.

## 3. Results

### 3.1. Varied Mesiodens Phenotypes within the Hmong Family (Family 1)

**Figure 1:**
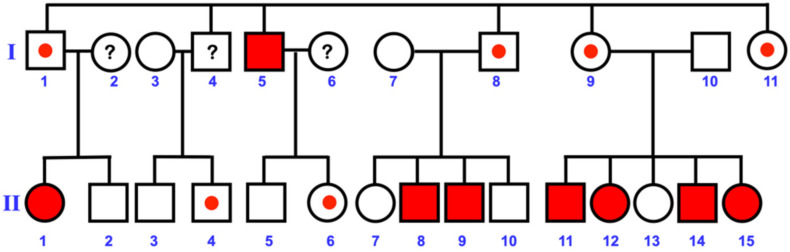
Pedigree of the two-generation Hmong family (Family 1). This family consists of 26 members, of which eight are affected with mesiodens (block red) and 18 are unaffected (white). Red dots represent non-penetrant individuals who have the variant p.Glu603Lys but not mesiodens. A number of the affected are children of the non-penetrant. Family members I-2, I-4, I-6, and II-1 were not available for genetic study. The phenotypes of the parents of I-1, I-4, I-5, I-8, I-9, and I-11 are unknown.

**Figure 2:**
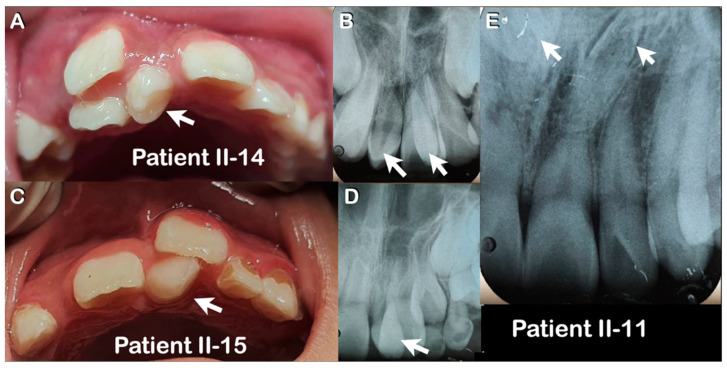
Clinical and radiographic pictures of mesiodens (arrows) in (**A**) Patient II-14. (**B**) Periapical radiograph in (**A**) shows double mesiodentes (arrows). (**C**) Patient II-15. (**D**) Periapical radiograph in (**C**) shows mesiodens. (**E**) Periapical radiograph of patient II-11. Note double inverted mesiodentes (arrows).

A two-generation large Hmong family living in the Wiang Kaen city of Chiang Rai province in Thailand with an age range from 7–62 years participated in the study. This family consisted of 26 members, of which eight were affected by mesiodens and 18 were unaffected (Figure 1). However, four members (I-2, I-4, I-6, and II-1) were not available for genetic study. Clinical and radiographic examinations were performed on all participants (Table 4). Of the eight members with mesiodens, six had erupted midline teeth (75%), while two remained unerupted (25%). Six (75%) had single mesiodens and two (25%) had double mesiodentes (Figure 2A,B). Two (25%) family members had inverted mesiodens (Figure 2E). This highlights the phenotypic variability in mesiodens formation even within the same family.

### 3.2. Whole Exome Sequencing, Sanger Direct Sequencing, and Bioinformatic Analysis

**Figure 3:**
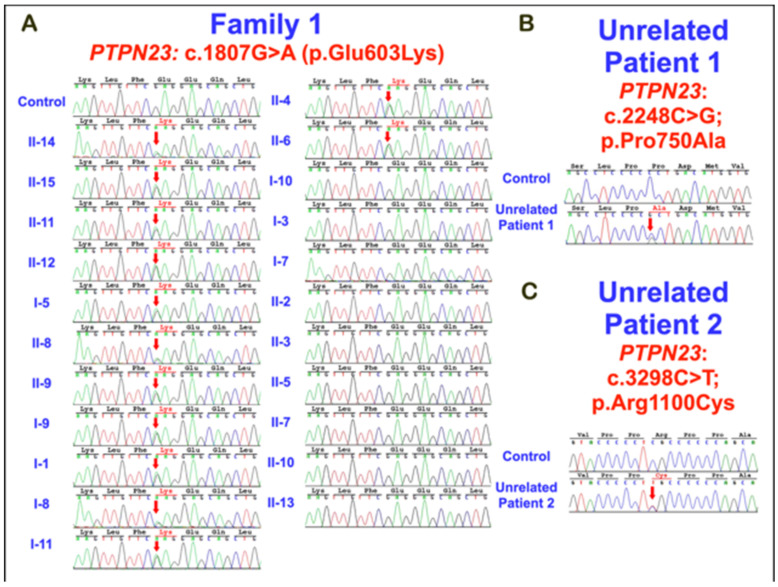
Chromatograms of the heterozygous missense (**A**) c.1807G>A; p.Glu603Lys, (**B**) c.2248C>G; p.Pro750Ala, and (**C**) c.3298C>T; p.Arg1100Cys mutations in *PTPN23* gene in family 1, unrelated patient 1 and unrelated patient 2, respectively.

Whole exome and Sanger direct sequencing revealed a missense variant in the *PTPN23* gene (chr3:g.47450916G>A; NM_015466.4:c.1807G>A; NP_056281.1:p.Glu603Lys; rs141113890) in all seven affected individuals analyzed (I-5, II-8, II-9, II-11, II-12, II-14, and II-15) and additionally in six unaffected relatives (I-1, I-8, I-9, I-11, II-4, and II-6). Six unaffected family members (II-2, II-3, II-5, II-7, II-10, and II-13) and three unaffected unrelated members (I-3, I-7, and I-10) did not carry the variant (Figure 1 and Figure 3, Table 4).

**Figure 4:**
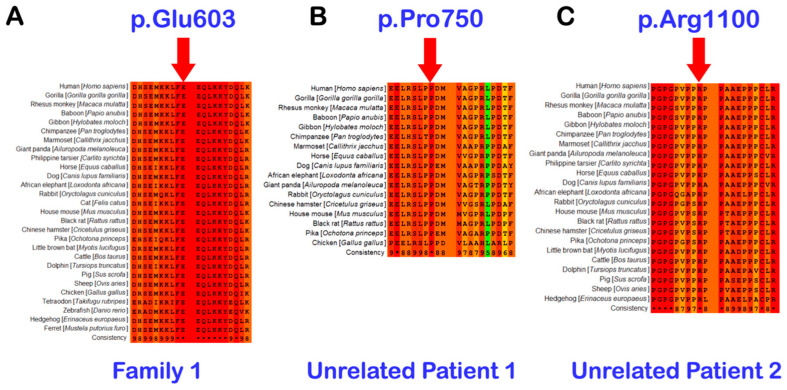
Conservation of amino acid residues (**A**) p.Glu603, (**B**) p.Pro750, and (**C**) p.Arg1100 across vertebrate species. All amino acids are highly conserved.

*PTPN23* c.1807G>A (p.Glu603Lys) is most likely the pathogenic variant in this family because it was the only variant that all eight affected family members had in common. Moreover, this variant is extremely rare in the general population. According to gnomAD, the variant is not seen in over 30,608 alleles in the South Asian, Finnish, Jewish, Latin American, and African American populations. It is seen in five of 18,372 alleles in the East Asian population (allele frequency = 0.00027) and four of 113,444 alleles in the European population (allele frequency = 0.00003526). In the general population, it is seen in only 10 of 250,702 alleles, with an allele frequency of 0.00003989 and without any homozygous variation. The mutation is predicted as disease-causing with a probability of 0.99973 (MutationTaster), possibly damaging with a score of 0.488 (PolyPhen-2) and tolerated with a score of 0.516 (SIFT). The combined annotation-dependent depletion (CADD) score, a tool for scoring the deleteriousness of single nucleotide variants, of the p.Glu603Lys variant is 22.5, suggesting that this variant is predicted to be among the 1.0% most deleterious possible substitutions in the human genome (https://genome.ucsc.edu accessed on 8 January 2022). The multiple alignments of *PTPN23* amino acid presented that the amino acid Glu603 is highly conserved in many vertebrate species (Figure 4).

### 3.3. Unrelated Mesiodens Patients Identified with Rare PTPN23 Variants

**Figure 5:**
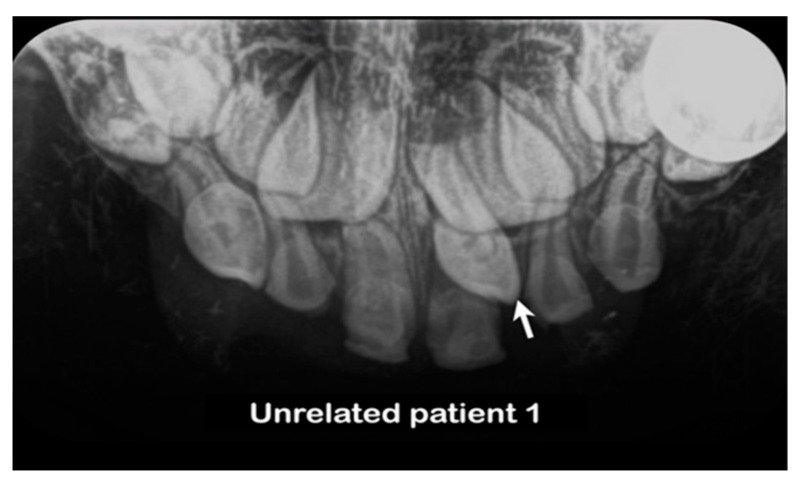
Periapical radiograph of mesiodens of the unrelated patient 1 (arrow).

Having identified the c.1807G>A; p.Glu603Lys variant in *PTPN23* was the likely pathogenic variant in family 1 by WES, we cross-checked our in-house exome bank of 720 people affected with various disorders in order to identify patients in our cohort who had rare *PTPN23* variants. We identified two rare variants in unrelated patients, both of whom displayed mesiodens. The chr3: g.47451536C>G; NM_015466.4: c.2248C>G; NP_056281.1: p.Pro750Ala (rs199549354) variant in the *PTPN23* gene was identified in an unrelated Thai patient 1 with mesiodens (Figure 3 and Figure 5, Table 4). According to gnomAD, the allele frequency of this variant is 0.001436 with 43 alternative alleles in a total of 29,938 alleles and two homozygotes in South Asians, allele frequency = 0.000 with none of the alternative alleles in a total of 19,702 alleles in East Asians and a total allele frequency = 0.0001639 with 45 alternative alleles in 274,536 alleles from global populations. This amino acid is conserved in various vertebrate species (Figure 4). The mutation is predicted as disease-causing with a probability of 0.99989 (MutationTaster), possibly damaging with a score of 0.888 (PolyPhen-2) and damaging with a score of 0.016 (SIFT).

**Figure 6:**
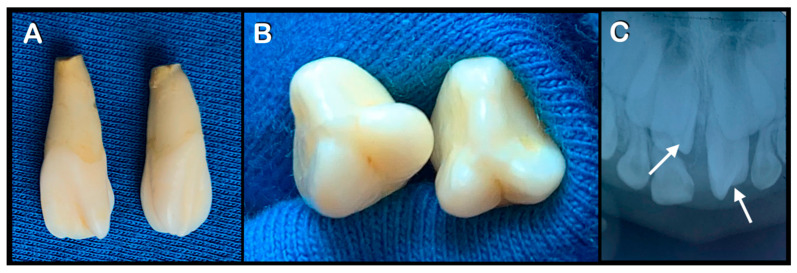
Unrelated patient 2. Extracted double mesiodentes in (**A**) Frontal view. (**B**) Top view. (**C**) Periapical radiograph prior to extraction (arrows). Note screwdriver-shaped crown morphology of both mesiodentes.

The chr3: g.47452586C>T; NM_015466.4: c.3298C>T; NP_056281.1: p.Arg1100Cys (rs201946361) variant was identified in the second unrelated Thai patient 2 with double mesiodentes (Figure 3 and Figure 6, Table 4). According to gnomAD, the allele frequency of this variant in the general population is 0.0006730, with 169 alternative alleles in a total of 251,122 alleles and no homozygotes in the East Asian population. In the South Asian population, six alleles were found in a total of 27,774 alleles with an allele frequency of 0.0002160. The mutation is predicted as a polymorphism with a probability of 0.99999 (MutationTaster), possibly damaging with a score of 0.946 (PolyPhen-2) and damaging with a score of 0.003 (SIFT). The CADD scores of p.Pro750Ala and p.Arg1100Cys variants are 20.8 and 20.3, respectively, suggesting that these variants are predicted to be among the 1.0% most deleterious possible substitutions in the human genome (https://genome.ucsc.edu accessed on 8 January 2022).

### 3.4. PTPN23 and Its Relationship to EGFR during Early Murine Tooth Development

**Figure 7:**
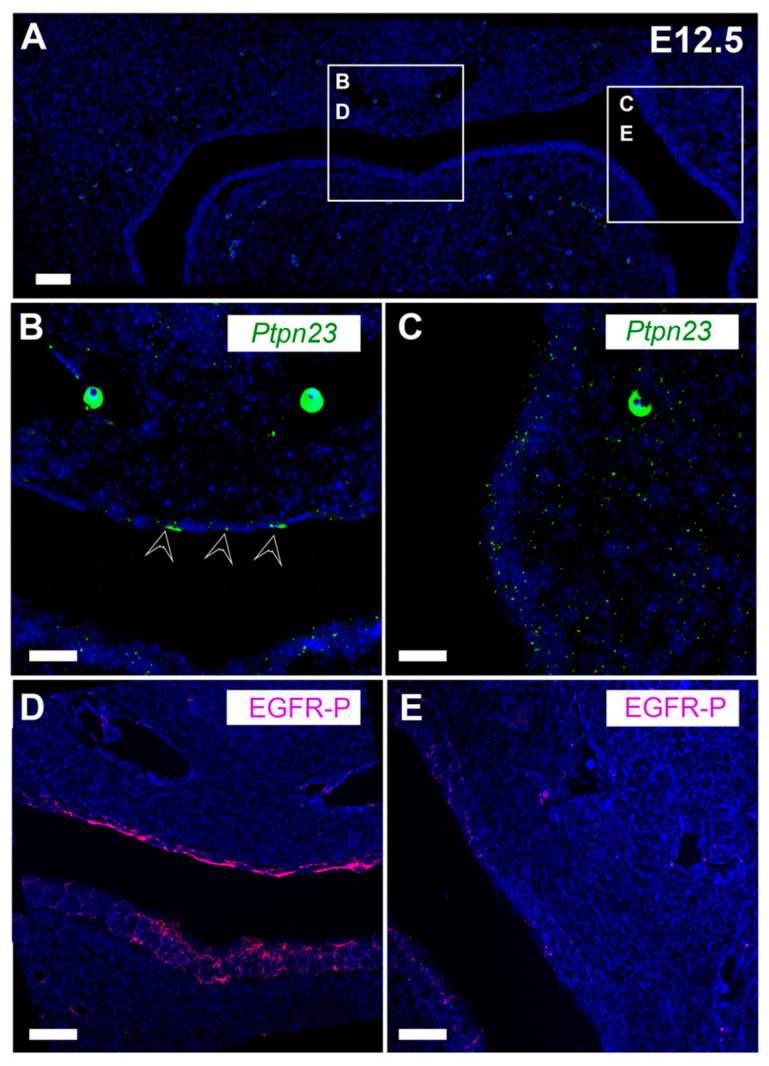
Expression of *PTPN23* and EGFR in the oral cavity. Murine E12.5 frontal sections at the midline at the upper incisors. Serial sections used for *PTPN23* and EGFR. (**A**) DAPI (blue) stained section highlighting regions shown in (**B**–**E**). (**B**,**C**) *PTPN23* RNAscope. Signal shows up as green dots. Large circular patches are autofluorescence from blood cells. (**D**,**E**) Immunofluorescence EGFR protein in pink. (**B**) *PTPN23* is expressed in the midline epithelium (arrows) and (**C**) robustly in the more lateral epithelium and mesenchyme. (**D**) EGFR is expressed in the midline in the forming incisor region, similar to *PTPN23* expression. (**E**) EGFR is weakly expressed more laterally. Scale bar in (**A**): 100 μm. Scale bar in (**B**–**E**): 50 μm.

**Figure 8:**
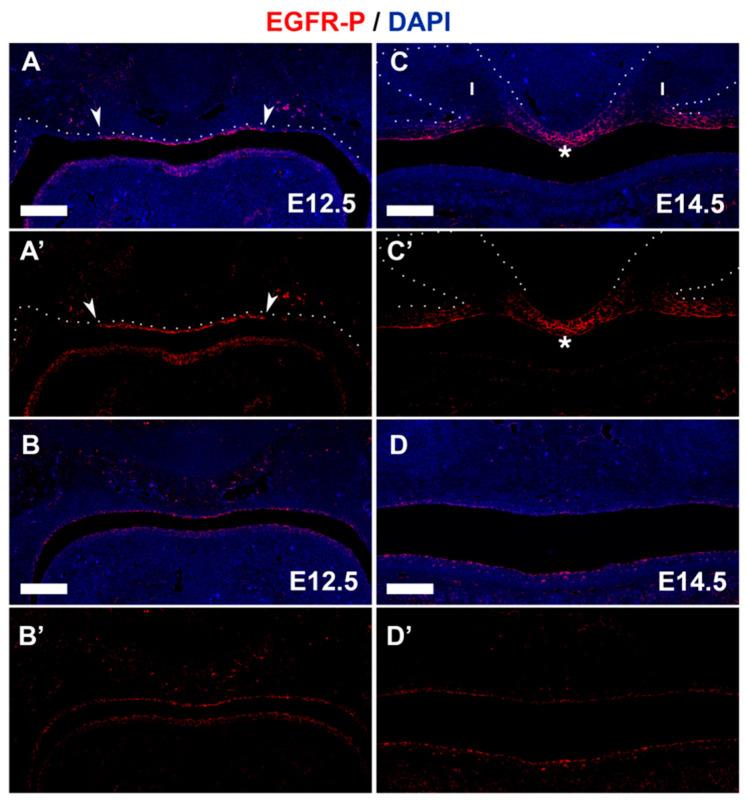
EGFR is expressed at high levels in the forming midline. Murine frontal sections. (**A**–**B’**) E12.5. (**C**–**D’**) E14.5. (**A**–**D**) Immunofluorescence EGFR (pink/red) plus DAPI (blue). (**A’**,**B’**,**C’**,**D’**) EGFR only. (**A**,**A’**,**C**,**C’**) Anterior region. (**B**,**B’**,**D**,**D’**) More posterior region. Arrows in (**A**,**A’**) highlight border of expression of EGFR in the midline at E12.5. Asterix in (**C**,**C’**) highlights midline between cap stage incisors (outlined with white dots). I = incisor. Scale bar in (**A**,**C**): 100 μm (same scale in all images).

To understand how changes in *PTPN23* function might impact tooth development, we analyzed its expression during tooth development in the mouse using RNAscope. Previously a LacZ reporter has been used to analyze the expression of *PTPN23* in the developing mouse embryo, with robust expression in the brain, vertebrae, and submandibular salivary glands [58]. At E12.5, *PTPN23* was observed in the midline oral epithelium and higher levels, more laterally in both the epithelium and mesenchyme (Figure 7A,C, green dots). *PTPN23* is a known regulator of endosomal trafficking and functions to deactivate membrane receptors, such as EGFR [59], with mutations in *PTPN23* in patients potentially impacting EGFR signaling due to the availability of the receptor. The expression of EGFR protein was, therefore, followed in the mouse during the early stages of tooth development by immunofluorescence using serial sections to those used for *PTPN23*. Interestingly, EGFR was expressed at high levels in the midline epithelium at E12.5 (Figure 7D and Figure 8A,A’), with a reduced expression more laterally and further back in the mouth (Figure 7E and Figure 8B,B’). At E14.5, when the incisor tooth germs are at the cap stage, EGFR retained its high expression in the midline between the incisors, with a lack of expression in the main body of the incisors (Figure 8C,C’) and further back in the oral cavity (Figure 8D,D’). EGFR was, therefore, expressed in the midline, where mesiodens teeth originate.

### 3.5. Decreased Phosphatase Activity of Mutant PTPN23

**Figure 9:**
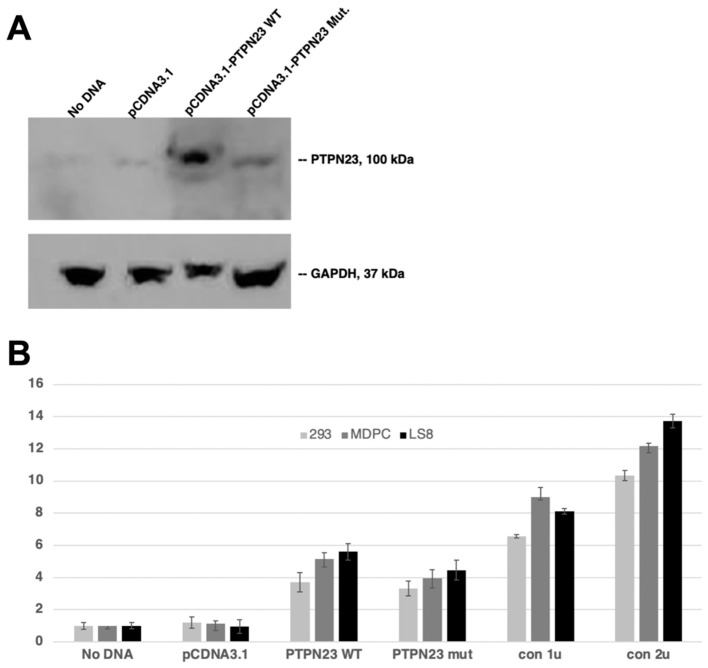
*PTPN23* mutant protein has reduced phosphatase activity. (**A**) Cell lysates from transfected and untransfected cells were probed for *PTPN23* expression. Both untransfected and control transfected cells showed low levels of endogenous *PTPN23* protein expression. WT and mutant *PTPN23* transfected lysates showed increased expression (n = 3). (**B**) Phosphatase activity was measured after no transfection and transfection of control DNA, WT *PTPN23,* and mutant *PTPN23* (Glu603Lys). Three different cell lines were used to measure phosphatase activity: HEK293, MDPC (dental pulp cells), and LS-8 (oral epithelial cells). WT *PTPN23* has low phosphatase activity in the three cell lines, compared to Potato Acid Phosphatase controls with either 1 unit (u) or 2 units. However, mutant *PTPN23* has decreased phosphatase activity compared to WT *PTPN23* (n = 3).

To understand if the mutation in *PTPN23* affected protein stability, equal amounts of transfected lysates were analyzed for protein expression levels. Interestingly, untransfected and empty vector-transfected cells showed little *PTPN23* endogenous protein expression (Figure 9A). HEK293 cell lysates transfected with wild type (WT) and mutant *PTPN23* showed an increase in *PTPN23* protein expression compared to controls, with the c.1807 G>A; p.Glu603Lys; rs141113890 mutation potentially influencing protein stability (Figure 9A). To determine if the mutation affected phosphatase activity, we analyzed both WT and mutant proteins in three different cell lines (HEK293, MDPC dental pulp cells, and LS-8 oral epithelial cells). As expected, the no transfection and empty vector transfection controls showed limited phosphatase activity, while transfected WT *PTPN23* led to an approximately five- to six-fold increase in phosphatase activity (Figure 9B). Importantly, mutant *PTPN23* phosphatase activity was decreased compared to WT (Figure 9B). However, both the WT and mutant *PTPN23* proteins demonstrated less activity than 1 unit of Potato Acid Phosphatase, used as a positive control (Figure 9B).

## 4. Discussion

A mesiodens is a supernumerary tooth located in the midline of the premaxilla. It has been reported to be associated with Nance–Horan syndrome [41,42,43,44] and holoprosencephaly [45]. Nance–Horan syndrome (NHS; MIM 302350) is a rare X-linked developmental disorder caused by mutations in *NHS*. Clinical findings of the patients include congenital cataracts, intellectual disability, autism, and dental anomalies, including screwdriver blade-shaped incisors, supernumerary teeth, and mesiodens [41,42,43,44,60].

A number of research groups have tried to find the gene responsible for the development of isolated mesiodens. Unfortunately, this had not been successful, likely because most cases of mesiodens are sporadic. Even though familial cases have been reported, the number of those affected was not enough to locate the gene [61]. One of the major obstacles in trying to find the gene responsible for mesiodens is its well-known non-penetrance of inheritance (approximately 50% penetrance). This means that if you see a child with mesiodens, other family members may carry the same mutation even though they do not have mesiodens. In addition, a large number of cases (53.8-78.8%) of mesiodentes do not erupt into the oral cavities (Table 2) [3,5,6,7,8,9]. Without radiographic examination, those with unerupted mesiodens might have mistakenly been considered unaffected. These problems have made gene hunting for mesiodens phenotypes complicated and unsuccessful. However, recently genetic variants in *LRP4*, *LRP5*, *LRP6*, *WLS,* and *DKK1* have been reported to be implicated in mesiodens with or without oral exostoses, including torus palatinus, torus mandibularis, and buccal exosteses [46,47,48,49,50]. We were very fortunate to meet a large Hmong family living in Chiang Rai, a province at the border of Thailand and Myanmar. This family, comprised of 26 members, had eight affected and 18 unaffected individuals. Mesiodens were found to be more common in males than females, with a ratio of 1.7:1. The morphology of mesiodens of the patients was not classified because some of them were already extracted, and some were unerupted. To our best knowledge, this is the largest family affected by mesiodens that has been reported in the literature.

Whole exome and Sanger direct sequencing identified the heterozygous missense c.1807G>A; p.Glu603Lys, c.2248C>G; p.Pro750Ala, and c.3298C>T; p.Arg1100Cys variants in the *PTPN23* gene as the molecular etiologies of the mesiodens phenotypes in family 1, unrelated patient 1, and unrelated patient 2, respectively. For family 1, the mode of inheritance was autosomal dominance with incomplete penetrance. Many affected were the children of non-penetrant parents. Of the 13 family members analyzed who carried the mutation, only seven were found to have mesiodens; therefore, the penetrance was 53.84%. Despite the family having the same variant, the mesiodens phenotype (erupted/unerupted, inverted/normal, single/double) in those affected was highly variable, highlighting that other factors must impact these differences. We were not able to report the morphology of the mesiodentes found in the Hmong family because most of their mesiodentes were extracted, and some of them were unerupted. It was, therefore, impossible to know the exact morphology without using three-dimension computed tomography.

*PTPN23* is located in chromosome 3p21.3 and is composed of 25 exons. It encodes a 1636 amino acid non-receptor protein tyrosine phosphatase type 23 (PTPN23) or His-domain protein tyrosine phosphatase (HDPTP) [62,63]. Bi-allelic mutations in *PTPN23* in humans are associated with autosomal recessive neurodevelopmental disorder and structural brain anomalies with or without seizures and spasticity (NEDBASS; MIM 618890) [62] and autosomal recessive microcephalic complex hereditary spastic paraplegia [64]. In keeping with this, *PTPN23* is expressed in the cerebral cortex, thalamus, and hypothalamus of adult mice and in the embryonic nervous system [59,65]. Unfortunately, mesiodens or other dental anomalies were not mentioned in these studies, and unerupted mesiodens could have been missed [62,64]. Our patients with mono-allelic *PTPN23* mutations were healthy with no neuromuscular disorders. Evidently, the results of our study and the previous reports of patients with neuromuscular disorders [62,64] suggest that the phenotypes of patients with *PTPN23* mutations depend on the context of the mutations and/or other factors, including modifying genes.

**Figure 10:**
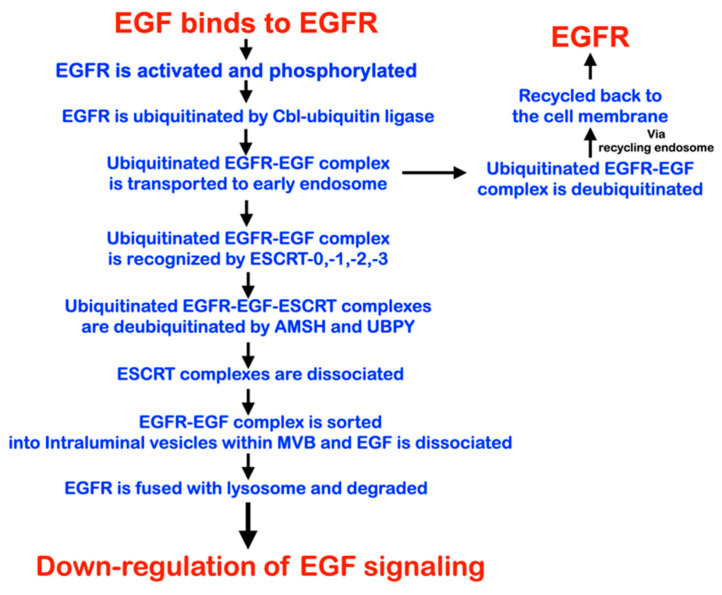
EGFR–Multivesicular Biogenesis–Lysosomal Degradation pathway.

**Figure 11:**
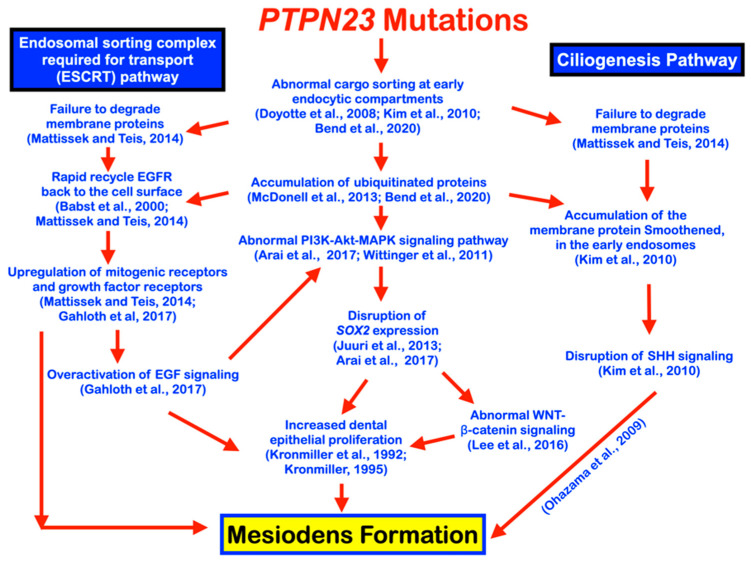
Proposed pathogenetic pathways as a result of *PTPN23* mutations that lead to mesiodens formation. Refs: [59,62,70,72,73,77,78,79,80,81,82,83,84,85].

*PTPN23* is a catalytically inactive phosphatase that binds to tyrosine-phosphorylated proteins in order to prevent them from dephosphorylation [66,67]. Homozygous *PTPN23* knockout mouse embryos display an accumulation of ubiquitinated proteins in endosomes, disruption of the MVB biogenesis, smaller body size, significant malformations, and subsequent embryonic lethality at E8.5 [58,59,62,68]. The mouse embryos, therefore, die prior to any signs of tooth development. The function of *PTPN23* is restricted to the early endosome at the initiation of the ESCRT–MVB pathway, where it binds to ESCRT-0 to downregulate ubiquitinated cargoes and promotes forward movement of receptors from the early endosome towards the lysosomes, thereby leading to downregulation of the signal [68,69,70] (Figure 10). *PTPN23* is crucial for releasing EGFR from ESCRT-0 and allowing it to engage ESCRT-III [71]. Aberrant interaction between *PTPN23* and ESCRT-0 is expected to result in enhanced recycling of endocytosed EGFR [72,73,74] and subsequent overactivation of EGFR and MAPK signaling [73,75]. Depletion of *PTPN23* has been shown to cause the accumulation of ubiquitin–protein conjugates in tubulo-vesicular endosomal compartments and reduction of EGFR sorting to endosomal lumen [59,64,71] (Figure 10 and Figure 11). The decision for EGFR to be recycled to the cell membrane and retain its function or sorted to the endosomes for subsequent lysosomal degradation depends on the strength of the signals [74,76] (Figure 10).

**Figure 12:**
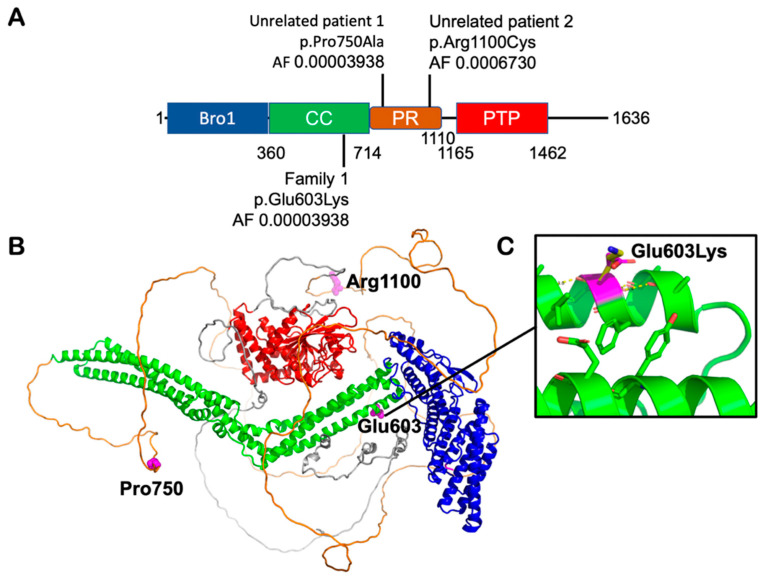
Organization of *PTPN23* domains and location of the variants. (**A**) Residue numbers indicate domain boundaries, and arrows show locations of the mutations found in our patients. CC: coiled-coil domain; PR: proline-rich region; PTP inactive phosphatase. (**B**) Theoretical Alpha fold model of the *PTPN23* protein structure. Colors as in (**A**). The mutated residues are highlighted in magenta as sphere models. (**C**) Close-up view of the Glu603 wild-type residues (magenta stick model) and its lysine substitution (yellow).

*PTPN23* comprises several functional domains, including the Bro1 domain, the coiled-coil (CC) domain, a long proline-rich region (PR), and the inactive protein tyrosine phosphatase (PTP) domain [62,77] (Figure 12). The p.Glu603Lys mutation, which was found in family 1, is located in the CC domain (Figure 12). Glu603 is solvent exposed and its substitution to a lysine is not perturbing the structure

However, the substitution of a negatively charged glutamic acid with a positively charged lysine would affect the electrodynamics of this region. Given that the CC is a scaffolding domain, such a change in surface charge may influence its intra- or intermolecular interactions. The p.Glu603Lys mutation may affect protein stability and function [62,77]. Alternatively, non-structural effects (linked to splicing or translation, for example) are possible. The p.Pro750Ala and p.Arg1100Cys mutations, identified in unrelated patients 1 and 2, respectively, are located in the unstructured and flexible PR (Figure 12). Even though these variants will not impact the structural integrity or stability of the protein, they may affect post-translational modifications (PTMs) or ligand binding sites. To date, no PTMs have been reported in the vicinity of these three mutations, but PR motifs are known to bind to SH3 or WW domains. The *PTPN23* CC has indeed been shown to bind to the EGFR adaptor protein Grb2 [78]. Therefore, collectively, these mutations are predicted to disrupt EGFR signaling by hampering protein–protein interactions.

We have provided evidence that the c.1807 G>A; p.Glu603Lys; rs141113890 *PTPN23* mutation associated with family 1 may affect protein stability. According to a Western blot, transfected mutant *PTPN23* cell lysates had less protein expression compared to WT transfected cell lysates (Figure 9A). In addition, a lower level of *PTPN23* phosphatase activity was shown in all mutant transfected cell lines compared to WT transfected cell lines (Figure 9B). The results suggest that either less expression and/or decreased activity of mutant *PTPN23* protein activity could be causative of the mesiodens in the patients.

*PTPN23* mutations in our patients are predicted to result in the accumulation of ubiquitin–protein conjugates in vesiculotubular endosomal compartments and reduction of EGFR sorting to the endosomal lumen, decreased degradation of EGFR and subsequent overactivation of EGFR signaling (Figure 11) [59,78]. *Egf* is expressed in developing jaws immediately before the formation of the dental lamina [79,80]. In culture, the addition of *Egf* has been demonstrated to result in both inhibitions of normal tooth formation and induction of ectopic supernumerary teeth in the diastema [79,80]. Here, we show that while *PTPN23* has a relatively broad expression domain in the murine oral cavity, its target EGFR is expressed at high levels in the forming midline. Given the restricted expression of EGFR, and the ability of ectopic EGF signaling to generate ectopic teeth, this pathway is a likely target for mesiodens formation (Figure 7 and Figure 8).

Notably, patients with bi-allelic variants in *NHS* and *PTPN23* share phenotypes, including developmental brain disorders, intellectual disability, cataracts, and autism [62,81]. The presence of mesiodens in patients with *PTPN23* variants and patients with *NHS*-associated Nance–Horan syndrome [41,42,43,44] raises a question if there is an association between the functions of NHS and *PTPN23*. The role of NHS is to maintain the integrity of the actin at the cell membrane, which is important for cell shape, migration, and intercellular junction [82,83,84]. Actin plays an important role in producing force to form an endosome [85]. As previously mentioned, *PTPN23* binds with ESCRT-0 to encourage the forward movement of ubiquitinated EGFR endosomes toward the lysosomes, leading to the downregulation of the signal [68,69]. Therefore, mesiodens formation in patients with *NHS* or *PTPN23* mutations might relate to the disruptive endocytosis process.

It is hypothesized that disruption of EGFR signaling, as a result of *PTPN23* mutations and subsequent decreased mutant *PTPN23* phosphatase activity, would lead to disruption in the expression of the transcription factor *SOX2* [86,87] via the PI3K–Akt signaling pathway [86,88] (Figure 11). The finding of mutations in the phosphatase gene (PTPRH) resulting in aberrant EGFR activity supports our hypothesis [89]. The PI3K–Akt signaling pathway is likely to be involved in mesiodens pathogenesis because *Sox2*-positive odontogenic epithelial stem cells have been demonstrated to contribute to supernumerary tooth formation [87,90] and mutations in *SOX2* have been reported to be associated with syndromic supernumerary teeth in SOX2 anophthalmia syndrome [91,92]. Sox2 is crucial for the initiation of tooth formation and regulates the progenitor state of dental epithelial cells [87,93,94,95,96,97]. The *SOX2* lineage has been shown to give rise to successional teeth [87]. Sox2 disrupts Wnt signaling by binding to β-catenin, a central regulator of the Wnt signaling pathway [94]. The Wnt–β-catenin signaling pathway is known to be crucial for tooth development and overactivation of Wnt/β-catenin signaling results in supernumerary tooth formation or odontoma [96] (Figure 11). The association of genetic variants in Wnt/β-catenin signaling pathway and mesiodens formation [46,47,48] suggests the mutations in *PTPN23* in our patients were upstream of the Wnt/β-catenin signaling pathway in the pathogenetic process.

Additionally, abnormal BMP signaling may be involved in mesiodens patients with *PTPN23* mutations because mutations in *PTPN23* may lead to disruptive ESCRT recruitment, MVB sorting, degradation of BMP receptors, hyperactivation of BMP signaling, overactivation of WNT signaling, and mesiodens formation [98].

Recycling endosomes are a dynamic vesiculotubular compartment exporting endocytosed membrane proteins and lipids to the cell surface via vesicular intermediates [99,100]. The endocytic recycling pathway has also been associated with ciliogenesis. *PTPN23* has a specific role in ciliary vesicle targeting. Silencing of *PTPN23* has been shown to significantly reduce the number of ciliated cells [99]. Knockdown *PTPN23* has been demonstrated to result in the accumulation of the transmembrane protein Smoothened in early endosomes [99]. It is hypothesized that mutations in *PTPN23* would result in abnormal endocytic trafficking and accumulation of Smoothened in the early endosomes, leading to a disruption of SHH signaling [101], which could contribute to mesiodens formation (Figure 11). Disruption to a number of signaling pathways driven by *PTPN23* mutations could, therefore, account for the formation of mesiodens.

### Limitations of the Study

Mesiodens are frequently unerupted, and therefore, their incidence can be hidden. In our study, it was not possible to use cone beam computed tomography (CBCT) on each patient due to the additional exposure to radiation, but this would have provided more information on the morphology of the mesiodens observed. We used mouse embryos for gene and protein expression, presuming conservation of the pathway in mammals, but human embryonic and fetal tissue would have beneficial. Finally, it was not possible to test the impact of the loss of function of *PTPN23* in the mouse as the null mice are early lethals; therefore, conditional models would need to be generated.

## 5. Conclusions

In conclusion, *PTPN23* is a regulator of endosomal trafficking, and its function is to move activated membrane receptors forward from ESCRT-0 towards ESCRT-III [59], thereby regulating the activity of a number of signaling pathways. We show that mutations in *PTPN23* are associated with the formation of midline upper supernumerary teeth, known as mesiodens. We hypothesize that these mutations disrupt the accumulation of activated EGFR and other signaling pathways in early endosomes, leading to abnormal signaling at the early stages of tooth development and subsequent supernumerary tooth formation.

## Figures and Tables

**Table 1:** The prevalence of mesiodens in different populations.

Authors	Ethnic	Number of Patients	Prevalence (%)	Radiographic Examination	Male:Female
Thilander and Myrberg, 1973 [2]	Swedish	6398	0.9	yes	Not mentioned
von Arx, 1992 [14]	Swiss	Not mentioned	Not mentioned	yes	2.6:1
Asaumi et al., 2004 [13]	Japanese	Not mentioned	Not mentioned	yes	2.8:1
Gunduz et al., 2008 [3]	Turkish	23000	0.3	yes	2.1:1
Kositbowornchai et al., 2010 [4]	Thai	570	1.05	yes	Not mentioned
Mukhopadhyay, 2011 [5]	Indian	7932	0.8	yes	1.78:1
Kazanci et al., 2011 [6]	Turkish	3351	0.3	yes	1.5:1
Lara et al., 2013 [7]	Brazilian	1995	1.5	yes	1.5:1
Ramesh et al., 2013 [10]	Indian	Not mentioned	Not mentioned	yes	2.05:1
Goksel et al., 2018 [8]	Turkish	1902	5.04	yes	1.9:1
Aren et al., 2018 [9]	Turkish	58142	0.1	yes	Not mentioned

**Table 2:** Characteristics of mesiodens.

Authors	N (Patients)	N (Teeth)	Number	%	Unerupted %	Orientation	%
Asaumi et al., 2004 [13]	200	256	Single	73	Not mentioned	Vertical	27
			Double	26		Inverted	67
			>3	1		Transverse	6
Gunduz et al., 2008 [3]	69	85	Single	76.8	78.8	Vertical	55.2
			Double	23.1		Inverted	37.6
						Transverse	7
Mukhopadhyay, 2011 [5]	64	78	Single	78.1	53.8	Vertical	62.8
			Double	21.9		Inverted	30.8
						Transverse	6.4
Kazanci et al., 2011 [6]	10	12	Single	80	66.7	Vertical	58.3
			Double	20		Inverted	33.3
			>3	0		Transverse	8.4
Lara et al., 2013 [7]	30	36	Single	80	75	Vertical	75
			Double	20		Inverted	13.9
			>3	0		Transverse	11.1
Goksel et al., 2018 [8]	101	130	Single	76.23	78.46	Vertical	63.1
			Double	18.81		Inverted	20
			>3	4.95		Transverse	16.9
Aren et al., 2018 [9]	59	83	Single	61	68.7	Vertical	77.1
			Double	37.3		Inverted	22.9
			>3	1		Transverse	0

**Table 3:** Published reports of familial occurrence of mesiodens.

Types of Familial Occurrences	References	Descriptions
Siblings	Sedano and Gorlin, 1969 [19]	2 siblings affected (normal parents)
	Cadenat et al., 1977 [20]	Discordant dizygotic twin (parents’ phenotype: N/A)
	Almeida et al., 1995 [11]	3 siblings affected (parents’ phenotype: N/A)
	Desai and Shah, 1998 [21]	2 siblings affected (parents’ phenotype: N/A)
	Marya and Kumar, 1998 [22]	2 siblings affected (parents’ phenotype: N/A)
	Gallas and Garcia, 2000 [23]	2 siblings affected (parents’ phenotype: N/A)
	Takahashi et al., 2017 [24]	2 siblings affected (parents’ phenotype: N/A)
Monozygotic twins	Hunstadbråten, 1965 [28]	Mirror imaged discordant
	Schön, 1974 [29]	Concordant
	Bucci and Martina, 1975 [30]	Discordant
	Carton and Rees, 1987 [31]	Mirror imaged discordant
	Choi et al., 1990 [32]	Near concordant
	Beere et al.,1990 [33]	Mirror imaged discordant
	Seddon et al., 1997 [34]	Near concordant
	Brand et al., 2000 [35]	Mirror imaged discordant
	Townsend et al., 2005 [36]	8 pairs of discordant
	Sharma, 2008 [37]	Discordant
	Babacan et al., 2010 [38]	Concordant
	Gurgel et al., 2013 [39]	Concordant
	Reddy et al., 2013 [40]	Discordant
More than one generation	Mason and Rule, 1995 [25]	Father, mother and 2 sons
	Sharma, 2003 [26]	Father and daughter
	Severin et al., 2009 [27]	Family 1: Mother, 2 daughters, uncle, and cousin
		Family 2: Father, daughter, and son
		Family 1: Mother and son
	Takahashi et al., 2017 [24]	Family 2: Father and son
		Family 3: Mother and 3 sons
Skip generation	Severin et al., 2009 [27]	Grandmother and grandson

**Table 4:** Clinical and molecular findings of patients and their family members (NM_015466.4; NP_056281.1).

Families	Patients	Ethnic	Gender	Phenotypes	Orientation	Eruption	*PTPN23* Mutations
Family 1	II-14	Hmong	Male	Double mesiodentes	Normal	Erupted	c.1807G>A; p.Glu603Lys; rs141113890
	II-15	Hmong	Female	Single mesiodens	Normal	Erupted	
	II-11	Hmong	Male	Double mesiodentes	Inverted	Unerupted	
	II-12	Hmong	Female	Single mesiodens	Inverted	Unerupted	
	I-5	Hmong	Male	Single mesiodens	Normal	Erupted	
	II-8	Hmong	Male	Single mesiodens	Normal	Erupted	
	II-9	Hmong	Male	Single mesiodens	Normal	Erupted	
	II-1	Hmong	Female	Single mesiodens	Normal	Erupted	Not available
	I-9	Hmong	Female	Unaffected	-	-	c.1807G>A; p.Glu603Lys; rs141113890
	I-1	Hmong	Male	Unaffected	-	-	
	I-8	Hmong	Male	Unaffected	-	-	
	I-11	Hmong	Female	Unaffected	-	-	
	II-4	Hmong	Male	Unaffected	-	-	
	II-6	Hmong	Female	Unaffected	-	-	
	I-10	Hmong	Male	Unaffected	-	-	No mutation
	I-3	Hmong	Female	Unaffected	-	-	
	I-7	Hmong	Female	Unaffected	-	-	
	II-2	Hmong	Male	Unaffected	-	-	
	II-3	Hmong	Male	Unaffected	-	-	
	II-5	Hmong	Male	Unaffected	-	-	
	II-7	Hmong	Female	Unaffected	-	-	
	II-10	Hmong	Male	Unaffected	-	-	
	II-13	Hmong	Female	Unaffected	-	-	
			Male: Female with mesiodens = 1.67:1 (5:3)	Single: Double mesiodens cases = 6:2 (3:1)	Inverted mesiodens cases = 25% (2/8)	Unerupted mesiodens cases = 25% (2/8)	Penetrance = 53.84% (7/13)
Family 2	Unrelated patient 1	Thai	Female	Single mesiodens	Normal	Erupted	c.2248C>G p.Pro750Ala rs199549354
Family 3	Unrelated patient 2	Thai	Male	Double mesiodentes	Normal	Erupted	c.3298C>T p.Arg1100Cys rs201946361

## Data Availability

Not applicable.
